# Joint Effects of Physical Activity and BMI on Risk of Hypertension in Women: A Longitudinal Study

**DOI:** 10.1155/2014/271532

**Published:** 2014-01-23

**Authors:** Caroline Jackson, Gerrie-Cor Herber-Gast, Wendy Brown

**Affiliations:** ^1^Centre for Longitudinal and Life Course Research, School of Population Health, University of Queensland, Herston Road, Brisbane, QLD 4006, Australia; ^2^School of Human Movement Studies, University of Queensland, Blair Drive, St. Lucia, Brisbane, QLD 4072, Australia

## Abstract

*Introduction*. There is debate as to whether physical activity counteracts the adverse effect of weight on health outcomes. We investigated how physical activity modifies the effect of body mass index (BMI) on hypertension risk. *Methods*. BMI, physical activity, and hypertension were measured at baseline and at three-year interval for 14 years (from 1996 to 2010), in 10,339 participants in the Australian Longitudinal Study on Women's Health. Generalised estimating equation models for binary repeated measures were performed to determine the individual and joint effects of BMI and physical activity on incident hypertension. *Results*. At baseline (mean age 47.6 ± 1.5 SD), 57% were healthy weight, 28% overweight, and 14% obese. Increasing BMI and decreasing physical activity were associated with increased risk of hypertension. Physical activity attenuated the positive association between weight and risk of hypertension, especially for obese women. Compared to healthy weight high active women, risk of hypertension in obese high active women was 3.4 times greater (OR 3.43, 95% CI 2.68, 4.39) and in obese inactive women 4.9 times greater (OR 4.91, 95% CI 3.92, 6.13). *Conclusions*. Both physical activity and maintenance of a healthy body weight are associated with lower risk of hypertension. Physical activity reduced but did not remove the effect of obesity on hypertension risk.

## 1. Introduction

There is persuasive evidence that obesity increases [1], whilst regular physical activity reduces the risk of cardiovascular disease [2]. However, contradictory findings from previous studies has led to debate about the relative importance of weight and physical activity with respect to risk of future adverse health outcomes [3–5]. Two studies have shown that the risk of all-cause mortality among overweight but fit men was similar to [6] or less than [7] the risk among healthy weight unfit men, leading to the hypothesis that cardiovascular fitness may offset the health risks of being overweight.

In contrast, physical activity, rather than cardiovascular fitness, has not been found to negate the excess mortality or cardiovascular risk resulting from being overweight or obese [8–12]. In keeping with this, there is evidence that physical activity also does not completely counteract the risk of diabetes among overweight or obese individuals [3]. However, little is known about the effect of physical activity on the association between weight and hypertension [5]. To our knowledge, only one previous prospective population-based study has addressed this issue [13]. In it, Hu et al. found that among women, physical activity attenuated the increased risk of hypertension associated with higher weight, but there was little difference in risk between overweight physically active women and normal weight physically inactive women. However, BMI and physical activity were measured at one time point only, and the authors did not distinguish between overweight and obese participants. As both physical activity and BMI are likely to change over time, and the effects of physical activity may be different for overweight and obese women, we aimed to investigate relationships between both physical activity and BMI and risk of hypertension and the extent, if any, to which physical activity moderates the effect of BMI on risk of incident hypertension among mid-aged participants in the Australian Longitudinal Study on Women's Health.

## 2. Methods

### 2.1. Study Setting

We included participants from the Australian Longitudinal Study on Women's Health (ALSWH), a national population-based study of women born in 1921–26, 1946–51, and 1973–78. Women were randomly selected from the Medicare database, which covers all citizens and permanent residents of Australia, including refugees and immigrants. Women born in 1946–51, and thus aged 45–50 at baseline, were first surveyed, using self-completed questionnaires, in 1996 (survey 1, S1) and again in 1998 (survey 2, S2), 2001 (survey 3, S3), 2004 (survey 4, S4), 2007 (survey 5, S5), and 2010 (survey 6, S6). Full details of the recruitment and response rates are reported elsewhere [14].

### 2.2. Study Population

For this study we included women from the 1946–51 cohort who reported no history of hypertension at baseline and were not underweight at baseline or at followup. We included those who responded to at least two sequential surveys and who had complete data on all exposures and hypertension at the appropriate time points.

### 2.3. Exposures and Outcome

At each survey, BMI was computed as self-reported weight (kg)/height (m^2^) and categorized as healthy weight (18.5–24.9 kg/m^2^), overweight (25–29.9 kg/m^2^), or obese (≥30 kg/m^2^). At S1, physical activity scores were derived from reported frequency and intensity of activity. The weekly frequency of exercise (never = 0; once a week = 1; 2 or 3 times per week = 2.5; 4–6 times per week = 5; every day = 7; and more than once a day = 10) was multiplied by the intensity of activity (vigorous = 5 (e.g., aerobics, jogging) and less vigorous = 3 (e.g., walking and swimming)). The resulting physical activity scores ranged from 0 to 50 and were categorized as none (<5), low (5 to <15), moderate (15 to <25), or high (≥25). At S2 to S6, physical activity was assessed using modified (self-report) version of the Active Australia questionnaire [15]. The women were asked to report frequency and total duration of walking, moderate, and vigorous intensity leisure time physical activity during the last week. A physical activity score in metabolic equivalent (MET) minutes per week was derived using the following formula: MET·min/week = (walking minutes ∗ 3.5 METs) + (moderate minutes ∗ 4.0 METs) + (vigorous minutes ∗ 7.5 METs). Physical activity was categorized as sedentary (0–39 metabolic equivalent (MET)  min/wk), low (40–599 MET min/wk), moderate (600–1199 MET min/wk), and high (≥1200 MET min/wk), based on international guidelines [16]. We created a composite variable of BMI and physical activity, thereby creating 12 categories of each possible BMI/physical activity combination at each survey. Smoking at each survey was categorised as never, ex-smoker, or current smoker. Alcohol intake was categorized as non/rarely drinker (less than once per month); low risk (up to 14 drinks per week); or risky drinkers (15 or more drinks per week) [17]. Education, ascertained at S1, was categorised as no formal qualifications; school certificate; trade/apprenticeship; or higher education. At S1 women were asked if they had ever been diagnosed with or treated for diabetes mellitus, heart disease, stroke, or hypertension. At subsequent surveys they were asked if they had been diagnosed with these conditions in the three-year period since the previous survey. Women were classified as having diabetes if they reported having been diagnosed with or treated for diabetes by a doctor. A history of cardiovascular disease was defined as present if women reported having been diagnosed with or treated for heart disease or stroke by a doctor. New cases of hypertension were identified through self-report at S2 to S6.

### 2.4. Statistical Analyses

To account for multiple observations for each participant, we used generalised estimating equation regression models for binary outcome data (using an unstructured correlation structure and a logit link function). We calculated odds ratios (ORs) with 95% confidence intervals (CIs) for the association between each of BMI and physical activity (included as time-varying covariates) and incident hypertension. Women who reported hypertension at any survey did not contribute to the analyses of time periods thereafter. We calculated crude ORs before adjusting for age and education at baseline and history of cardiovascular disease, diabetes, smoking, and alcohol, as time-varying covariates. Once women reported cardiovascular disease or diabetes, they were considered to have that condition at subsequent surveys. We examined the joint association between BMI and physical activity by comparing the odds of hypertension in each combined BMI/physical activity category with the odds in the reference category of healthy weight and high physical activity. We also performed a sensitivity analysis, where we repeated the primary analysis but excluded the women with a history of diabetes or CVD at baseline.

This paper was prepared in accordance with the Strengthening the Reporting of Observational Studies in Epidemiology (STROBE) statement [18].

## 3. Results

**Figure 1:**
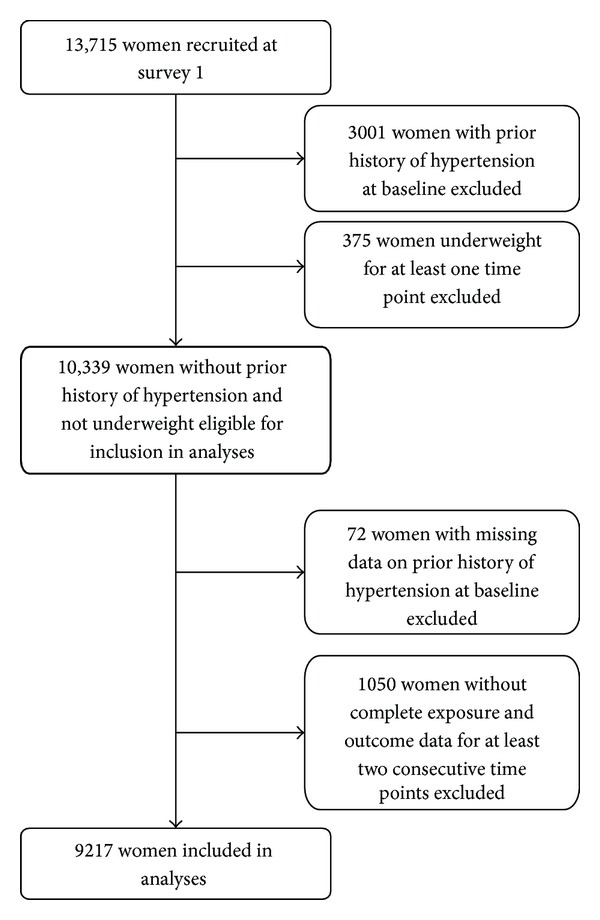
Flow diagram showing included and excluded participants.

Of 13,715 women recruited at baseline, 3001 had a previous history of hypertension and 375 were underweight at one or more time points. Of the remaining women, 72 did not respond to the question on hypertension at baseline and 1050 did not have complete data on all exposures and the outcome for at least two consecutive surveys. Therefore, data from 9217 (89%) women were included in the analyses (Figure 1). In general, women who were excluded due to nonreturn of surveys or incomplete data were less healthy and had a lower education level than those who were included in the analyses (Table 1). There was no difference between included and excluded women in terms of BMI at baseline, but a greater proportion of excluded women were sedentary.

At baseline, the mean age of included women was 47.6 (±1.5) years and 5097 (56.5%) were of healthy weight. A further 2596 (28.8%) were overweight and 1325 (14.7%) obese. Almost a fifth of all women were highly physically active, a quarter were moderately active, a third reported low physical activity, and a quarter were classified as participating in no physical activity. As shown in Table 2, we compared baseline characteristics of women in the three weight categories at baseline.

### 3.1. Individual Effects of BMI and Physical Activity on Hypertension Risk

During followup, 2260 incident hypertension cases were identified, giving a hypertension prevalence of 24.5% in 2010. The odds of developing hypertension were almost double (adjusted OR 1.92, 95% CI 1.72, 2.15) in overweight women and more than three times higher (adjusted OR 3.52, 95% CI 3.12, 3.97) in obese women than in healthy weight women (*P* for trend <0.001; Table 3).

The effect of physical activity on hypertension risk was less marked, but still evident. Compared with high active women, odds of hypertension were not significantly higher in those who reported moderate activity, but 26% higher in those who reported low physical activity (adjusted OR 1.26, 95% CI 1.11, 1.43) and 28% higher in those who reported no physical activity (adjusted OR 1.28, 95% CI 1.11, 1.47, *P* for trend <0.001; Table 3).

### 3.2. Joint Effects of BMI and Physical Activity on Hypertension Risk

**Figure 2:**
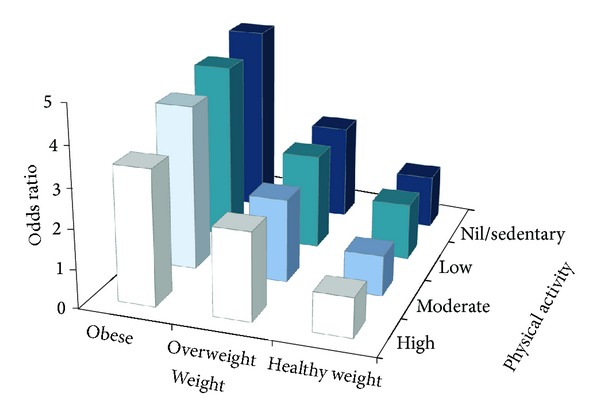
Joint associations of weight and physical activity with hypertension.

A formal test for interaction between BMI and physical activity revealed a significant interaction (*P* value < 0.0001). The odds of hypertension were 37% higher in healthy weight inactive women than in healthy weight high active women (adjusted OR 1.37, 95% CI 1.05, 1.78; Table 4). Compared with healthy weight women, overweight and obese women also had increased odds of hypertension, irrespective of activity level (Table 4; Figure 2). However, physical activity reduced the effect of BMI on hypertension, and this was more apparent for obese than for overweight women. The odds of hypertension were 3.4 times higher in obese high active women (adjusted OR 3.43, 95% CI 2.68, 4.39) and 4.9 times higher in obese inactive women (adjusted OR 4.91, 95% CI 3.92, 6.13) than in healthy weight high active women (Table 4). Physical activity therefore reduced but did not remove the effect of obesity on hypertension risk (Figure 2). We found similar results in a sensitivity analysis where we excluded women with a history of diabetes or CVD at baseline (data not shown).

## 4. Discussion

In this population of mid-aged women, both high BMI and low physical activity were individually associated with an increased risk of hypertension, but the effects were more marked for BMI than for physical activity. When the joint associations of BMI and physical activity with hypertension were studied, we found that being active attenuated but did not eliminate the excess risk of hypertension associated with being overweight or obese. Odds of hypertension were almost twice as high in women who were obese but physically active as in healthy weight inactive women.

### 4.1. Comparisons with Other Studies

Our results are consistent with previous findings on the joint association of obesity and physical activity with diabetes [3], cardiovascular morbidity, and mortality [8–12]. All these studies found that BMI and physical activity independently predicted incidence of diabetes and CHD, but that physical activity did not completely counteract the risk among overweight or obese individuals. Similarly, one study found that multiple low-risk lifestyle factors were significantly associated with lower risk of hypertension among normal weight and overweight women, but not among obese women [19].

Until now, only one prospective study had examined the joint associations of BMI and physical activity with incidence of hypertension [13]. Similar to our findings, this study showed that physical activity attenuated the increased risk of hypertension associated with weight. However, this study showed little difference in risk between overweight active women and normal weight inactive women. In contrast, we found that the risk of hypertension was higher in obese active women than in healthy weight inactive women. Our findings therefore suggest that even high levels of physical activity cannot completely negate the increased risk of hypertension associated with overweight and obesity. Differences in findings may be explained in part by the diversity of hypertension, physical activity, and BMI measures. We used a self-reported measure of hypertension, whereas the previous study defined hypertension on the basis of national data for hypertension treatment. Furthermore, physical activity in the previous study included occupational, commuting, and leisure time physical activity, whereas our measure did not include occupational activity. However, unlike our study, the previous study collected data on exposures at baseline only and any changes in exposure during followup were therefore not accounted for. This is important because BMI typically increases with age, especially during mid-age. Also, in our cohort activity levels increased when women retired from paid work [20]. Effect estimates from the previous study are therefore likely to be residually confounded. Furthermore, obese individuals were not differentiated from overweight individuals. This is important because the risk of hypertension was much higher and the effects of activity more marked in the obese women in our study. It has been suggested that moderate to high levels of cardiovascular fitness, in contrast to physical activity, completely eliminate the increased mortality or cardiovascular risk associated with obesity [6, 7]. In the present study we did not assess physical fitness. However, a recent study of the association between hypertension and cardiorespiratory fitness found that the adverse contribution of baseline BMI to the risk of hypertension was substantially attenuated, but not eliminated, after controlling for cardiorespiratory fitness [21]. However, this study was small and the findings need to be replicated in larger prospective studies.

### 4.2. Limitations

There are a few limitations to our study. First, exposures and incident hypertension are limited by self-report. However, self-reported height and weight have been shown to be valid for calculating BMI in this cohort [22]. The physical activity questionnaire in this study has also been found to have measurement properties which compare favourably with those of other commonly used physical activity measures [23]. Moreover, studies that compared self-reported hypertension against medical records, indicated good validity of self-reported hypertension [24, 25]. A validation study found that 86% of self-reported hypertension cases were verified by medical records [25]. In addition, the prevalence of self-reported hypertension is also in line with the worldwide hypertension prevalence in women [26]. It is also likely that any misclassification will have occurred at random, which may have attenuated the observed findings toward the null, leading to an underestimate of effect estimates. We also relied on self-report of previous history of cardiovascular disease. Concordance between self-report of cardiovascular disease with health records has been found to be substantial [25]. Some misclassification may have occurred, but again this is more likely to have occurred at random. Second, although we adjusted for a range of major lifestyle, biological and psychosocial risk factors for hypertension, residual confounding due to unmeasured factors, such as diet (including salt intake) and presence of prehypertension, cannot be excluded. Third, our study population included mid-aged women and thus we cannot necessarily extrapolate our results to women outside this age group or to men. Further study is therefore warranted in more diverse populations. Finally, we assessed adiposity through BMI measurement. Although BMI is correlated with percentage body fat [27], other clinical measures of adiposity, such as waist circumference or waist hip ratio, may be a better predictor of hypertension and should be examined in future studies.

### 4.3. Strengths

Our study has a number of strengths. To our knowledge, this study is one of the first to examine the combined effects of BMI and physical activity on incidence of hypertension. Our study population was large and community-based which allows us to extrapolate our findings to other middle-aged women. A further strength is the long duration of followup and subsequent large number of accrued hypertension cases, leading to sufficient power for assessing the joint effects of physical activity and BMI. The longitudinal approach of the ALSWH, with repeated measures of exposures and confounders, enabled us to model BMI and physical activity more accurately over time and to take into account changes in exposure variables during followup. Furthermore, the large study sample allowed us to differentiate between obese and overweight women.

### 4.4. Conclusions

Both elevated BMI and reduced physical activity appear to play an important role in the development of hypertension. The lowest odds of hypertension were in healthy women who reported moderate or high levels of physical activity, thus reinforcing the importance of maintaining a healthy weight and being physically active to reduce the risk of hypertension in mid-aged women, who often find weight reduction difficult. Risk of hypertension associated with overweight and obesity was however reduced considerably by increased physical activity levels. Overweight and obese women should therefore be encouraged to be more physically active. Further large prospective studies in both men and women, with more objective measures of BMI, physical activity, and hypertension, along with measurement of cardiorespiratory fitness, are needed to confirm our findings.

## Figures and Tables

**Table 1:** Baseline characteristics of included and excluded women.

Characteristic	Included *N* = 9217 *n* (%)	Excluded* *N* = 1122 *n* (%)	*P*-value
Age, mean years (±SD)	47.6 (±1.5)	47.6 (±1.5)	0.137
Marital status			
Married/de facto	7749 (84.3)	834 (75.5)	<0.001
Divorced/separated/widowed	1142 (12.5)	222 (20.4)	
Single	294 (3.2)	48 (4.4)	
Education			
No qualifications	1520 (16.5)	290 (28.2)	<0.001
School certificate	4444 (48.2)	506 (49.3)	
Trade/apprenticeship/higher education	3253 (35.3)	231 (22.5)	
BMI (kg/m^2^)			
18.5–25 (healthy)	5097 (56.5)	483 (52.9)	0.14
25–29 (overweight)	2596 (28.8)	283 (31.0)	
≥30 (obese)	1325 (14.7)	147 (16.1)	
Physical activity			
High	1546 (16.9)	174 (16.0)	0.001
Moderate	2394 (26.1)	238 (21.9)	
Low	2809 (30.6)	324 (29.8)	
Nil/sedentary	2420 (26.4)	350 (32.2)	
Smoking			
Never	4824 (53.7)	442 (44.2)	<0.001
Ex-smoker	2597 (28.9)	287 (28.7)	
Current	1571 (17.5)	270 (27.0)	
Alcohol intake			
Low risk	4679 (51.0)	431 (40.1)	<0.001
None	1280 (14.0)	202 (18.8)	
Rarely	2750 (30.0)	385 (35.8)	
Risky	457 (5.0)	57 (5.3)	
History of diabetes	175 (1.9)	36 (3.4)	0.001
History of cardiovascular disease	193 (2.2)	28 (8.7)	<0.001

*Women excluded due to missing data.

**Table 2:** Characteristics of study population at baseline, by baseline weight category.

Characteristic	Healthy weight (*N* = 5097) *n* (%)	Overweight (*N* = 2596) *n* (%)	Obese (*N* = 1325) *n* (%)
Age, mean years (± SD)	47.5 (±1.4)	47.6 (±1.5)	47.6 (±1.5)
Marital status			
Married/de facto	4253 (83.7)	2217 (85.8)	1115 (84.4)
Divorced/separated/widowed	667 (13.2)	289 (11.1)	158 (11.9)
Single	160 (3.2)	79 (3.1)	47 (3.6)
Education			
No qualifications	677 (13.3)	480 (18.5)	324 (24.5)
School certificate	2440 (47.9)	1262 (48.6)	655 (49.4)
Trade/apprenticeship/higher education	1980 (38.9)	854 (32.9)	346 (26.1)
Physical activity*			
High	970 (19.1)	402 (15.5)	154 (11.7)
Moderate	1397 (27.6)	664 (25.7)	278 (21.1)
Low	1509 (29.8)	809 (31.3)	435 (33.0)
Nil/sedentary	1191 (23.5)	713 (27.6)	451 (34.2)
Smoking			
Never	2680 (54.0)	1338 (52.7)	688 (53.0)
Ex-smoker	1397 (28.1)	786 (30.9)	375 (28.9)
Current	886 (17.9)	417 (16.4)	235 (18.1)
Alcohol intake^†^			
Low risk	2793 (55.1)	1276 (49.3)	512 (38.8)
None	642 (12.7)	375 (14.5)	233 (17.7)
Rarely	1362 (26.9)	811 (31.4)	516 (39.1)
Risky	268 (5.3)	124 (4.8)	58 (4.4)
History of diabetes	61 (1.2)	50 (1.9)	60 (4.5)
History of cardiovascular disease	99 (2.0)	56 (2.2)	33 (2.6)

*Physical activity defined as nil/sedentary (0–39 metabolic equivalent (MET) min/wk), low (40–599 MET min/wk), moderate (600–1199 MET min/wk), and high (≥1200 MET min/wk).

^†^Alcohol intake defined as low-risk (up to 14 drinks per week), none, rarely (any alcohol consumption less than once a month), and “Risky” (≥15 to 28 drinks per week).

**Table 3:** Odds ratios for the association between each of BMI and physical activity and incident hypertension.

	Unadjusted OR (95% CI)	*P* value for trend	Adjusted OR (95% CI)*	*P*-value for trend
Body mass index^†^ (kg/m^2^)				
Healthy	1.00 (reference)	<0.001	1.00 (reference)	<0.001
Overweight	1.95 (1.75, 2.18)		1.92 (1.72, 2.15)	
Obese	3.65 (3.25, 4.09)		3.52 (3.12, 3.97)	
Physical activity^‡^				
High	1.00 (reference)	<0.001	1.00 (reference)	<0.001
Moderate	1.13 (0.98, 1.29)		1.05 (0.92, 1.21)	
Low	1.43 (1.27, 1.61)		1.26 (1.11, 1.43)	
Nil	1.59 (1.39, 1.82)		1.28 (1.11, 1.47)	

*Adjusted for age and education at survey 1 and time-dependent smoking, alcohol consumption, presence of cardiovascular disease, physical activity (in the BMI analysis), and BMI (in the physical activity analysis).

^†^BMI (kg/m^2^) categorised as healthy: 18.5–25; overweight: 25–29; obese: ≥30.

^‡^Physical activity defined as nil/sedentary (0–39 metabolic equivalent (MET) min/wk), low (40–599 MET min/wk), moderate (600–1199 MET min/wk) and high (≥1200 MET min/wk).

**Table 4:** Odds ratios for the joint effect of BMI and physical activity on incidence of hypertension with underweight women excluded.

Weight^†^	Physical activity*			
	High	Moderate	Low	Nil
Unadjusted odds ratios (95% CI)				
Healthy weight	1.00 (reference)	1.07 (0.85, 1.35)	1.46 (1.19, 1.80)	1.38 (1.07, 1.79)
Overweight	2.22 (1.80, 2.74)	2.11 (1.69, 2.63)	2.50 (2.04, 3.06)	2.53 (2.01, 3.19)
Obese	3.47 (2.72, 4.42)	4.29 (3.39, 5.43)	4.61 (3.75, 5.66)	4.98 (4.01, 6.18)
Adjusted odds ratios^‡^ (95% CI)				
Healthy weight	1.00 (reference)	1.03 (0.81, 1.31)	1.45 (1.17, 1.79)	1.37 (1.05, 1.78)
Overweight	2.23 (1.80, 2.76)	2.13 (1.70, 2.67)	2.44 (1.98, 2.99)	2.46 (1.94, 3.12)
Obese	3.43 (2.68, 4.39)	4.21 (3.30, 5.37)	4.55 (3.69, 5.61)	4.91 (3.92, 6.13)

*Physical activity defined as nil/sedentary (0–39 metabolic equivalent (MET) min/wk), low (40–599 MET min/wk), moderate (600–1199 MET min/wk), and high (≥1200 MET min/wk).

^†^BMI (kg/m^2^) categorised as healthy: 18.5–25; overweight: 25–29; obese: ≥30.

^‡^Adjusted for age and education at survey 1 and time-dependent smoking, alcohol consumption, and presence of cardiovascular disease.
